# Nearly Complete Genome Sequences of Enterovirus 96 and Enterovirus 99 Strains Isolated in the Northern Region of Brazil

**DOI:** 10.1128/MRA.00549-19

**Published:** 2019-08-29

**Authors:** R. Scerni Machado, J. Lima Ferreira, J. C. Santos Alves, R. Silva Bandeira, P. Silva Lemos, L. Chaves Franco Filho, J. Ferreira Cardoso, J. L. Silva Gonçalves Vianez-Junior, F. Neto Tavares

**Affiliations:** a Enterovirus Laboratory, Virology Section, Evandro Chagas Institute, Brazilian Ministry of Health, Ananindeua, Pará, Brazil; b Postgraduate Program in Virology, Evandro Chagas Institute, Ananindeua, Pará, Brazil; c Center for Technological Innovation, Evandro Chagas Institute/SVS/MS, Ananindeua, Pará, Brazil; KU Leuven

## Abstract

In this study, we report the nearly complete genome sequences of one enterovirus 96 (EV-C96) isolate (strain 3499/BRA-PA/2010) and two enterovirus 99 (EV-C99) isolates (strains 3291/BRA-PA/2010 and 3944/BRA-PA/2011). The genetic characterization of different enterovirus strains allows for a better understanding of their molecular epidemiology and viral evolution.

## ANNOUNCEMENT

Enteroviruses are members of the *Enterovirus* genus and *Picornaviridae* family. These nonenveloped single-stranded RNA viruses have a genome of 7.5 kb and can infect both humans and animals. Human enteroviruses are ubiquitous agents transmitted from person to person via direct contact with gastrointestinal or upper respiratory tract secretions. Most infections are either asymptomatic or result in only mild symptoms like a nonspecific febrile illness or minor upper respiratory infection. However, enteroviruses can also cause a wide spectrum of illnesses, including hand-foot-and-mouth disease, acute hemorrhagic conjunctivitis, aseptic meningitis, acute flaccid paralysis, myocarditis, and severe neonatal sepsis-like disease (1).

The stool samples with strains 3291/BRA-PA/2010, 3499/BRA-PA/2010, and 3944/BRA-PA/2011, obtained from children with gastroenteritis, were received in 2010 and 2011 in Belém, Pará, Brazil, and processed at the Evandro Chagas Institute. Stool suspensions were inoculated into RD, HEp2, and L20B cell lines according to a WHO protocol for poliovirus detection (2). Cell cultures presenting cytopathic effect after 10 days (2 blind passages with 5 days of examination) were subject to the next-generation sequencing (NGS) protocol explained below.

Total RNA was extracted from 400 μl of the cell culture supernatant from each sample. We used the automated RNA extraction and purification protocol from the iPrep PureLink virus kit (Thermo Fisher Scientific). The cDNA double-stranded synthesis reaction was performed using the cDNA synthesis system kit (Roche Life Science). This kit synthesizes the cDNA in the following three steps: construction of the first strand using RNA as a template, synthesis of the second cDNA complementary strand, and, finally, cDNA purification. The cDNA was quantified using the Qubit double-stranded DNA (dsDNA) high-sensitivity (HS) assay kit (Thermo Fisher Scientific), and the integrity was checked with the 2100 Bioanalyzer instrument (Agilent).

The construction of the cDNA libraries was performed in two steps, namely, fragmentation of total RNA followed by synthesis of double-stranded cDNA, using a cDNA synthesis system kit (Roche) according to the manufacturer’s instructions. The workflow comprised the following three main steps: selection of fragments (size solution), ligation of adapters, and amplification phase by emulsion PCR (emPCR). Shotgun 454 reads were obtained on a Roche 454 GS-FLX+ genome sequencer with Titanium chemistry using standard protocols. Each sample was sequenced in an independent run. A total of 83,064 reads were generated for the sample 3499/BRA-PA/2010, 40,540 for 3291/BRA-PA/2010, and 52,600 for 3944/BRA-PA/2011. The average read lengths for samples 3499/BRA-PA/2010, 3291/BRA-PA/2010, and 3944/BRA-PA/2011 were 389, 358, and 331 bp, respectively. Reads shorter than 200 bp and with an average Phred score below 20 were removed. The remaining reads were assembled *de novo* using Newbler version 2.9 (parameters, minimum overlap of 40 bp, and minimum overlap identity of 90%). Contigs of viral origin were identified using BLASTX 2.2.31 against the nonredundant (nr) database. A total of 739, 20,707, and 2,294 reads were used in the assembly of the 3499/BRA-PA/2010, 3291/BRA-PA/2010, and 3944/BRA-PA/2011 genomes, respectively. Subsequently, the annotations of putative open reading frame (ORF) genes were predicted using Geneious 9.1.2 (Biomatters, New Zealand) (3). The assembled genomes of the strains 3291/BRA-PA/2010, 3499/BRA-PA/2010, and 3944/BRA-PA/2011 presented G+C contents of 44.4%, 43.7%, and 45% and lengths of 7,435, 7,454, and 7,438 bp, respectively, excluding the poly(A) tail. Complete coding sequences and partial 5′ noncoding regions (NCRs) were obtained for all three genomes. The 3291/BRA-PA/2010, 3499/BRA-PA/2010, and 3944/BRA-PA/2011 genomes each contain a single ORF which encodes a large viral polyprotein of 2,210, 2,219, and 2,210 amino acids, respectively.

**FIG 1:**
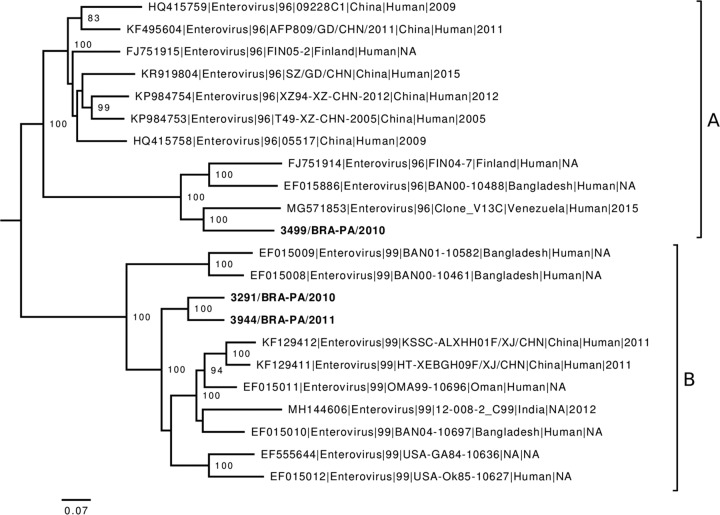
Maximum likelihood (ML) phylogenetic analysis of EV-C96 (clade A) and EV-C99 (clade B) strains. Nearly complete genome sequences of EV-C96 and EV-C99 strains available in GenBank were identified using BLAST and aligned with the sequences obtained in this study using MAFFT version 7.407 (4). The ML tree was generated with RaxML version 8.2.11 (5). A general time-reversible model with estimates for among site rate heterogeneity (GTRGAMMA) was applied. Node support was assessed using 1,000 bootstrap replicates. Midpoint rooting was applied for visualization. GenBank accession numbers are shown in the tip labels. Bold names indicate the strains sequenced in this study.

Phylogenetic analysis using complete coding genomic sequences grouped the samples 3291/BRA-PA/2010 and 3944/BRA-PA/2011 within the viral subtype enterovirus 99 (EV-C99), while the sample 3499/BRA-PA/2010 was classified as subtype enterovirus 96 (EV-C96) (Fig. 1). The strain 3499/BRA-PA/2010 showed the highest nucleotide identity (81.2%) with an isolate from Venezuela (GenBank accession number MG571853), while the strains 3291/BRA-PA/2010 and 3944/BRA-PA/2011 were highly similar (82.7% and 81.2%, respectively) to an isolate from Oman (GenBank accession number EF015011). The corresponding amino acid identities were 95.7% for 3291/BRA-PA/2010, 95.5% for 3944/BRA-PA/2011, and 94.7% for 3499/BRA-PA/2010.

This study reports the first nearly complete genome sequences of EV-C96 and EV-C99 strains isolated from Brazil that contributes genetic information that improves our understanding of the phylogenetic relationships of circulating enterovirus strains and highlights the importance of intensifying the use of molecular surveillance tools.

### Data availability.

EV-C96 and EV-C99 genome sequences were deposited in the GenBank (numbers MH484164 to MH484166) and SRA (accession number PRJNA551260) databases.
